# Verlängerung der Dosierungsintervalle von IL‐17‐ und IL‐23‐Inhibitoren bei erwachsenen Patienten mit Psoriasis: eine Pilotstudie aus der Praxis

**DOI:** 10.1111/ddg.15686_g

**Published:** 2025-06-11

**Authors:** Luca Mastorino, Paolo Dapavo, Michela Ortoncelli, Eleonora Bongiovanni, Pietro Quaglino, Simone Ribero

**Affiliations:** ^1^ Dermatologic Clinic Department of Clinical Medicine University of Turin Turin Italien

**Keywords:** Biologika, Deeskalation, IL‐17‐Inhibitoren, IL‐23‐Inhibitoren, Psoriasis, Verlängerung des Dosierungsintervalls, biologics, de‐escalation, dose spacing, IL‐17 inhibitors, IL‐23 inhibitors, Psoriasis

## Abstract

**Hintergrund und Ziele:**

Deeskalationsstrategien für Biologika bei der Behandlung von Psoriasis sind in der klinischen Praxis weit verbreitet. Die Verlängerung des Dosierungsintervalls beinhaltet die Deeskalation der Zeitspanne zwischen den Injektionen biologischer Arzneimittel.

**Patienten und Methoden:**

Hauptziele waren die Beschreibung der Trends bezüglich durchschnittlichem PASI, PASI 100, 90 und ≤ 1 vom Behandlungsbeginn bis 12 Monate nach Verlängerung des Dosierungsintervalls, die Analyse der Therapietreue bezüglich der Schemata mit verlängertem Dosierungsintervall und die gleichzeitige Beschreibung der phänotypischen Eigenschaften im Zusammenhang mit der Auswahl von Patientenkandidaten für eine therapeutische Verlängerung des Dosierungsintervalls. Es wurde eine Prä‐/Post‐Analyse hinsichtlich des durchschnittlichen PASI zum Zeitpunkt der Verlängerung des Dosierungsintervalls und bei Behandlungsbeginn sowie nach 3, 6, 9 und 12 Monaten im Anschluss an die Verlängerung des Dosierungsintervalls durchgeführt.

**Ergebnisse:**

Bei 61 der 1.144 mit IL‐23‐ oder IL‐17‐Inhibitoren behandelten Patienten wurde das Dosierungsintervall verlängert. Diese hatten zu Behandlungsbeginn einen niedrigeren durchschnittlichen Body‐Mass‐Index (BMI) (*p* = 0,011) und einen niedrigeren PASI (*Psoriasis Area Severity Index*) (*p* = 0,044) und hatten häufiger bereits Erfahrung mit Biologika (*p* = 0,033). 12 Monate nach der Verlängerung des Dosierungsintervalls erreichten 42,9%, 85,7% bzw. 92,9% der beobachteten Patienten PASI 100, 90 und ≤ 1. Es gab keine signifikanten Unterschiede beim durchschnittlichen PASI zwischen Beginn der Verlängerung des Dosierungsintervalls und nachfolgenden Zeitpunkten. Nach einem Jahr betrug die Therapietreue für das verlängerte Dosierungsintervall 70%.

**Schlussfolgerungen:**

Die Modulierung der Therapie wie die Verlängerung des Dosierungsintervalls ist bei den meisten Psoriasis‐Patienten eine wirksame Strategie, die zu einem Ansprechen mit läsionsfreier oder fast läsionsfreier Haut führt, das über längere Zeit erhalten bleibt.

## EINLEITUNG

Psoriasis ist eine entzündliche Hauterkrankung, die durch erythematöse, schuppige Plaques oder Flecken gekennzeichnet ist und die Streckseiten und palmoplantaren Oberflächen des Körpers, das Kapillitium und die Nägel betrifft.^1^ Weltweit leiden schätzungsweise 125 Millionen Menschen an Psoriasis. Dabei reicht die Prävalenz von 0,5% in asiatischen Ländern bis 8% in einigen europäischen Ländern. Eine leichte Form der Psoriasis kann mit externen Behandlungen wie topischen Kortikosteroiden, Vitamin‐D‐Analoga, Kombinationen aus Steroiden plus Vitamin‐D‐Analoga, Calcineurinhemmern und Keratolytika behandelt werden.^2^ Phototherapie (Schmalband‐UVB oder PUVA) kann bei mittelschwerer Psoriasis eine geeignete Option darstellen, während herkömmliche systemische Behandlungen wie Methotrexat, Ciclosporin und Acitretin nach wie vor häufig als Erstlinientherapien für mittelschwere bis schwere Psoriasis verwendet werden.^2^

Biologische Arzneimittel haben sich in den letzten beiden Jahrzehnten als hochwirksame und sichere Behandlung für mittelschwere bis schwere Psoriasis erwiesen.^3^, ^4^ In einer aktuellen Studie ermöglichten Interleukin(IL)‐17‐ und IL‐23‐Inhibitoren das Erzielen von früher als unvorstellbar geltenden Behandlungsergebnissen wie *Psoriasis Area Severity Index* (pASI) 90 und 100 sowie wichtige Auswirkungen auf die Lebensqualität wie *Dermatology Life Quality Index* (DLQI) 0/1.^3^ In den letzten Jahren gewann die Möglichkeit einer Modulation des Behandlungsschemas in Form einer Dosisreduktion (Deeskalation) oder Dosiserhöhung (Eskalation) an Bedeutung.^5^

Mögliche Deeskalationsstrategien sind die Verringerung der einzelnen therapeutischen Dosis, die Verringerung des mg/kg‐Verhältnisses, die Anzahl der Injektionen oder die Verlängerung des Dosierungsintervalls zwischen den Injektionen.^5^ Obwohl sie in Europa weit verbreitet sind, stellen Deeskalationsstrategien im Rahmen der Behandlung von Psoriasis eine Off‐Label‐Anwendung dar. Dagegen ermöglichen die Leitlinien zur Rheumatologie die Modulation einer biologischen Therapie gemäß dem klinischen Ansprechen.^5^ Darüber hinaus scheinen Erfahrungen aus der Praxis die Deeskalation bei anderen biologischen Behandlungen zu unterstützen, beispielsweise für Dupilumab bei schwerer atopischer Dermatitis.^6^, ^7^

Deeskalation bei Patienten mit dauerhaftem Ansprechen oder besonders gutem Ansprechen (Superresponder) könnte eine deutliche Verringerung der Gesundheitskosten ermöglichen, die sich zwar von Land zu Land unterscheiden, aber eine erhebliche Belastung für die Kosten im Gesundheitswesen von Ländern mit universalistischen nationalen Diensten sind.^5^ Außerdem könnte die Verringerung der Injektionen im Jahresverlauf die psychologische Belastung bei Psoriasis‐Patienten verringern, ihre Compliance verbessern und glaubhaft zu einer Verringerung der möglichen unerwünschten Ereignisse führen.^5^ Die Wirksamkeit unterschiedlicher Dosierungsschemata verschiedener biologischer Arzneimittel bei einer Population Behandlungs‐naiver Patienten wurde bereits in Phase‐II‐Studien untersucht.^8^, ^9^, ^10^ Studien aus der Praxis zur Deeskalation der Dosierung biologischer Arzneimittel bei Psoriasis‐Patienten konzentrieren sich vor allem auf Anti‐TNFα‐Therapien und zeigen unterschiedliche Ergebnisse.^11^ Derzeit liegen keine Daten zur Dosis‐Deeskalation, insbesondere zur Verlängerung des Dosierungsintervalls, für IL‐23‐ und IL‐17‐Inhibitoren vor.^12^, ^13^

Die vorliegende Studie hat das Ziel die Anwendbarkeit und Wirksamkeit der therapeutischen Modulation von IL‐17‐ und IL‐23‐Inhibitoren bei Psoriasis‐Patienten mit dauerhaftem Ansprechen auf diese Behandlungen aufzuzeigen.

## PATIENTEN UND METHODEN

Bei der vorliegenden Pilotstudie handelt es sich um eine Kohortenstudie mit retrospektiver Analyse der allgemeinen Eigenschaften und der Wirksamkeitsergebnisse von Psoriasis‐Patienten, die sich, egal aus welchen Gründen, einer Verlängerung des Dosierungsintervalls unterziehen, im Vergleich zu Patienten, die eine Biologikum‐Behandlung mit konventionellem Therapieschema erhalten. Alle Patienten über 18 Jahre, die mit biologischen Arzneimitteln – IL‐23‐Inhibitoren (Guselkumab, Risankizumab, Tildrakizumab), IL‐17‐Inhibitoren (Ixekizumab, Secukinumab, Brodalumab) und Anti‐TNFα‐Therapie (Adalimumab) – behandelt und an der *Dermatology Clinic* der *University of Turin* von Januar 2017 bis Dezember 2022 beobachtet wurden, wurden in die Studie aufgenommen.

*Primäre Ziele*:

1. Beschreibende Analyse der Trends hinsichtlich durchschnittlichem PASI, PASI 100, PASI 90 und PASI ≤ 1 bei Behandlungsbeginn (Datum des Therapiestarts des Biologikums), nach 16 Wochen, zum Zeitpunkt der Verlängerung des Dosierungsintervalls sowie 3, 6, 9 und 12 Monate nach der Verlängerung des Dosierungsintervalls bei den beobachteten Fällen.

*Sekundäre Ziele*:

1. Analyse der Therapietreue des Schemas mit verlängertem Dosierungsintervall.
2. Prä‐/Post‐Analyse hinsichtlich des durchschnittlichen PASI zum Zeitpunkt der Verlängerung des Dosierungsintervalls und bei Behandlungsbeginn sowie 3, 6, 9 und 12 Monate nach der Verlängerung des Dosierungsintervalls.

Außerdem beschreiben wir die phänotypischen Eigenschaften bezüglich der Auswahl der Patienten, die Kandidaten für eine therapeutische Verlängerung des Dosierungsintervalls waren, im Vergleich zur Population deren Behandlungsschema nicht modifiziert wurde. Die folgenden Aspekte wurden berücksichtigt: Durchschnittsalter, Population, deren Durchschnittsalter bei Beginn der Psoriasis, durchschnittlicher BMI, Geschlecht, Beteiligung schwer zu behandelnder Stellen (Kapillitium, Nägel, Genitalien, palmoplantare Region), Gelenkbeteiligung (*p*sA), Biologikum‐naiver Status, durchschnittliche Nachbeobachtungsdauer unter Behandlung mit herkömmlichen systemischen Medikamenten (Acitretin, Ciclosporin, Methotrexat), durchschnittlicher PASI und durchschnittlicher DLQI bei Einleitung der Biologikum‐Therapie.

### Zugelassene Dosierungsschemata

Herkömmliche, zugelassene Schemata für die berücksichtigten biologischen Wirkstoffe nach initialer, spezifischer Induktion (nähere Informationen finden sich im spezifischen Datenblatt):

- Adalimumab 40 mg subkutane (s.c.) Injektion (Inj.) alle 2 Wochen
- Ixekizumab 80 mg s.c. Inj. alle 4 Wochen
- Secukinumab 150 mg 2 s.c. Inj. alle 4 Wochen
- Brodalumab 210 mg s.c. Inj. alle 2 Wochen
- Guselkumab 100 mg s.c. Inj. alle 8 Wochen
- Risankizumab 75 mg 2 s.c. Inj. alle 12 Wochen (150 mg Fertigspritze zum Zeitpunkt der Studie nicht verfügbar)
- Tildrakizumab 100 mg s.c. Inj. alle 12 Wochen

### Statistische Analyse

Kontinuierliche Variablen wurden basierend auf der Verteilung jeder Variable als Mittelwert ± Standardabweichung (SD) oder als Median und Bereich beschrieben. Für kategorische Variablen wurden absolute und relative Häufigkeiten berichtet. Prozentwerte wurden auf die Anzahl nicht‐fehlender Werte basiert. Univariate lineare Regression (t‐Test für kontinuierliche Variablen und Chi‐Quadrat‐Test für kategorische Variablen), gefolgt von einem gemischten logistischen Regressionsmodell (für Werte mit p < 0,2), wurde eingesetzt, um potenzielle Faktoren zu untersuchen, die das Verlängern des Dosierungsintervalls beeinflussen können. Zur Analyse des Absetzens der verlängerten Dosierungsintervalle durch die Patienten im Rahmen der modulierten Dosierungsschemata wurden Methoden der Überlebensanalyse verwendet. Die Prä‐/Post‐Analyse wurde mit Hilfe einer univariaten linearen Regression (t‐Test) durchgeführt. Das Ereignis wurde als Absetzen der Dosiseskalation aus jeglichem Grund definiert, während die Beobachtungszeit vom Datum der Verlängerung des Dosierungsintervalls bis zum Termin der letzten Nachbeobachtung berechnet wurde. Die statistische Analyse wurde mit STATA 15.1 SE (StataCorp, 2017) durchgeführt. Alle Tests waren zweiseitig und die statistische Signifikanz wurde auf α = 0,05 festgelegt.

Die vorliegende Studie wurde von unserer Ethikkommission unter Protokoll SS‐Dermo‐20 genehmigt.

## ERGEBNISSE

Bei 61 von 1.144 Patienten (5,3%) wurde das Dosierungsintervall während der Therapie verlängert. Im Vergleich zu den 1.082 Patienten bei denen keine Deeskalation der Behandlung mit Biologikum vorgenommen wurde, zeigte die Population mit verlängertem Dosierungsintervall in der univariaten Analyse ein niedrigeres Alter (51 vs. 55 Jahre, p = 0,048), einen niedrigeren durchschnittlichen BMI (24,8 vs. 27,1, p < 0,001), einen niedrigeren durchschnittlichen PASI bei Beginn der Therapie mit Biologikum (12,2 vs. 14,5, p = 0,011) und waren häufiger Biologikum‐naiv (82,5% vs. 64,6%, p = 0,005). Keine und war häufiger Unterschiede zwischen den beiden Populationen gab es hinsichtlich des Alters bei Beginn der Psoriasis, der durchschnittlichen Zeit unter herkömmlichen systemischen DMARD (krankheitsmodifizierende Antirheumatika), mittlerem DLQI bei Beginn der Therapie mit Biologikum, Superresponder, Geschlecht sowie Beteiligung von schwierigen Stellen und Gelenken (Tabelle 1). In der multivariaten Analyse waren höherer BMI (OR 0,9, KI 0,83–0,98, p = 0,011), naiver Status (OR 0,26, KI 0,1–0,6, p = 0,003) und durchschnittlicher PASI bei Beginn der Therapie (OR 0,94, KI 0,88–0,99, p = 0,044) die einzigen drei Faktoren, die negativ mit einer Verlängerung der Behandlungsintervalle assoziiert waren. Dies widersprach teilweise den Ergebnissen der univariaten Analyse (Tabelle 1).

**TABELLE 1:** Univariate Analyse der allgemeinen Eigenschaften einer Population unter Dosis‐Deeskalation im Vergleich zu einer Population unter Standarddosis. Multivariate Analyse möglicher Patienteneigenschaften im Zusammenhang mit der Auswahl für ein Dosierungsschema mit Deeskalation.

	Verlängerter Dosierungsabstand	Standarddosis	p‐Wert
Gesamt n (%)	61 (5,3%)	1083 (94,7%)	0,602
Durchschnittsalter (SD)	51 (16)	55 (15,7)	**0,048**
Geschlecht (männlich) n (%)	38 (62,3%)	710 (65,6%)	0,602
Durchschnittlicher BMI (SD)	24,8 (3,4)	27,1 (5,5)	**< 0,001**
Durchschnittsalter bei Erkrankungsbeginn (SD)	36,8 (15,8)	34,9 (15,5)	0,396
Schwer zu behandelnde Bereiche n (%) (1039 Patienten)	39 (69,6%)	765 (77,8%)	0,155
PSA n (%) (1140 Patienten)	13 (22,8%)	316 (29,2%)	0,301
Biologikum‐naiv n (%) (1137 Patienten)	47 (82,4%)	695 (64,4%)	0,005
FU unter herkömmlichen systemischen Medikamenten Mittelwert (SD)	24,1 (21,2)	25,2 (31,5)	0,878
Durchschnittlicher PASI bei Behandlungsbeginn (SD)	12,2 (5,7)	14,5 (6,6)	**0,011**
Durchschnittlicher DLQI bei Behandlungsbeginn (SD)	20,9 (7,4)	22,5 (7,2)	0,156
Superresponder n (%)	17 (27,9%)	357 (33%)	0,409
Multivariate Analyse			

	*p*	*Odds‐Ratio*	*95%‐Konfidenzintervall*
Durchschnittsalter	0,338	0,99	0,97–1,01
Durchschnittlicher BMI	**0,011**	0,9	0,83–0,98
Schwer zu behandelnde Bereiche	0,989	0,99	0,44–2,23
Biologikum‐naiv	**0,003**	0,26	0,1–0,63
Durchschnittlicher PASI bei Behandlungsbeginn	**0,044**	0,94	0,88–0,99
Durchschnittlicher DLQI bei Behandlungsbeginn	0,198	0,97	0,93–1,01

IL‐Inhibitoren			Unterbrechung der Deeskalation
IL‐17‐Inhibitoren	41	67,2%	8
IL‐23‐Inhibitoren	20	32,8%	2
Brodalumab	20	32,8%	3
Ixekizumab	11	18%	3
Secukinumab	10	16,4%	2
Risankizumab	9	14,75%	0
Guselkumab	9	14,75%	2
Tildrakizumab	2	3,9%	0

	Behandlungsbeginn	16 Wochen	Tag der Deeskalation	3 Monate nach Deesk.	6 Monate nach Deesk.	9 Monate nach Deesk.	12 Monate nach Deesk.	Behandlungsbeginn vs. Deesk.	Deesk. vs. 3 Monate danach	Deesk. vs. 6 Monate danach	Deesk. vs. 9 Monate danach	Deesk. vs. 12 Monate danach
Durchschnittlicher PASI (SD)	12,2 (5,7)	1,6 (2,1)	0,7 (0,7)	1 (1,5)	1,2 (1,7)	1 (0,5)	0,6 (0,5)	< 0,001	0,209	0,07	0,19	0,899
PASI 100 (%)	0	34,6%	27,9%	31%	33,3%	25,9%	42,9%					
PASI 90 (%)	0	58,2%	73,8%	71,4%	69,4%	66,7%	85,7%					
PASI ≤ 1	0	61,8%	91,8%	83,3%	75%	81,5%	92,6%					

*Abk*.: SD, Standardabweichung; BMI, *Body‐Mass‐Index*; PSA, Psoriasis‐Arthritis; FU, Nachbeobachtung; PASI, *Psoriasis Area Severity Index*; DLQI, *Dermatology Life Quality Index*; IL, Interleukin; Deesk., Tag der Deeskalation

Verteilung der Patienten hinsichtlich der Klasse der Interleukin‐Inhibitoren und spezifische Behandlung. Anzahl und Anteil einer Unterbrechung der Dosis‐Deeskalation bei verschiedenen Behandlungen.

Durchschnittlicher PASI‐Trend bei beobachteten Patienten unter Deeskalation zu unterschiedlichen Zeitpunkten (Behandlungsbeginn, 16 Wochen, Tag der Deeskalation, 3, 6, 9, 12 Monate nach der Deeskalation). Prä‐/Post‐Analyse zwischen Behandlungsbeginn und Tag der Deeskalation sowie zwischen Tag der Deeskalation und nachfolgenden Zeitpunkten.

Erreichen von PASI 100, 90 und ≤ 1 zu jedem Zeitpunkt in beobachteten Fällen derselben Population.

Insgesamt betraf die Verlängerung des Dosierungsintervalls bei 41 Patienten einen IL‐17‐Inhibitor (67,2%) und bei 20 Patienten einen IL‐23‐Inhibitor (32,8%) (*p* = 0,036). Von Ersteren wurde bei 20 Patienten (32,8%) das Dosierungsintervall von Brodalumab verlängert: 15 Patienten passten die Dosierung zu 1 Fertigspritze 210 mg s.c. alle 3 Wochen an. Von diesen stellte ein Patient nach 6 Monaten auf 1 Fertigspritze s.c. alle 4 Wochen um. Fünf Patienten passten die Dosierung auf 1 Fertigspritze s.c. alle 4 Wochen an. Von diesen stellte ein Patient nach 6 Monaten auf 1 Fertigspritze s.c. alle 8 Wochen um. Neun Patienten (14,8%) verlängerten das Dosierungsintervall von Guselkumab, wobei alle die Dosierung auf 1 Fertigspritze 100 mg s.c. alle 12 Wochen anpassten. Von diesen stellte ein Patient aufgrund des Ansprechens nach 6 Monaten auf 1 Fertigspritze s.c. alle 16 Wochen um. Elf Patienten (18%) verlängerten das Dosierungsintervall von Ixekizumab, alle auf 1 Fertigspritze 80 mg s.c. alle 6 Wochen. Zehn Patienten (16,4%) verlängerten das Dosierungsintervall von Secukinumab auf 2 Fertigspritzen 150 mg s.c. alle 6 Wochen. Von diesen verlängerte ein Patient weiter auf 2 Fertigspritzen s.c. alle 8 Wochen. Zwei Patienten verlängerten das Dosierungsintervall von Risankizumab auf 75 mg, verabreicht als 2 Fertigspritzen s.c. alle 24 Wochen, und sieben Patienten auf 2 Fertigspritzen s.c. alle 16 Wochen. Zwei Patienten (3,3%) verlängerten das Dosierungsintervall von Tildrakizumab auf 1 Fertigspritze 100 mg s.c. alle 16 Wochen (Tabelle 1). Die Verlängerung des Dosierungsintervalls erfolgte im Durchschnitt 22,6 Monate (SD 15,2; Min. 3 bis Max. 70; Median 19,5; Q1–Q3 12–28,3) nach Einleitung der Behandlung mit dem entsprechenden Biologikum.

**ABBILDUNG 1:**
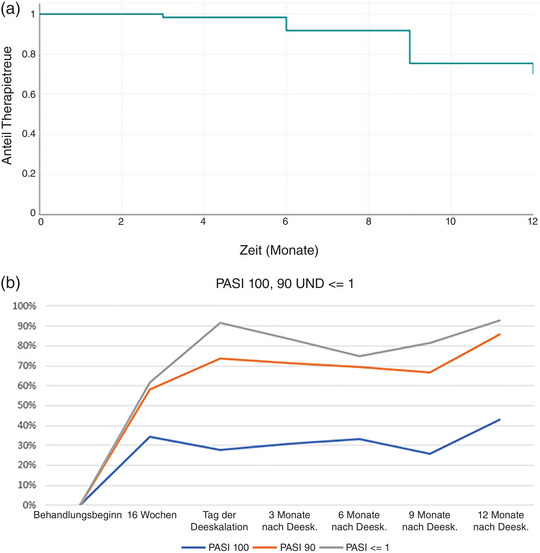
(a) Kaplan‐Meier‐Kurve zur Therapietreue nach 12 Monaten Dosierungsschema mit Deeskalation in der beobachteten Population. (b) Erreichen von PASI 100, PASI 90 und PASI ≤ 1 durch beobachtete Patienten nach 16 Wochen, am Tag der Deeskalation sowie nach den darauffolgenden 3, 6, 9 und 12 Monaten.

Insgesamt hatte sich bei den 61 Patienten, bei denen das Dosierungsintervall verlängert wurde, der anfängliche durchschnittliche PASI bei Beginn der Therapie mit voller Dosis nach 16 Wochen von 12,2 (SD 5,7) auf 1,6 (SD 2,1) verringert. Zum Zeitpunkt der Verlängerung des Dosierungsintervalls betrug der Wert 0,7 (SD 0,7) und blieb in den beobachteten Fällen bis zu 12 Monate nach Änderung der Dosis praktisch stabil (0,6, SD 0,8), mit einer leichten Verschlechterung nach 6 Monaten (1,2, SD 1,7). Zum Zeitpunkt der Verlängerung des Dosierungsintervalls hatten 27,9% PASI 100 erreicht. Dieser Wert verbesserte sich anschließend bei den beobachteten Fällen auf 33,3% nach 6 Monaten und auf 42,9% nach 12 Monaten. Nach 9 Monaten kam es zu einer leichten Verschlechterung des Outcomes (25,9%). Ein ähnlicher Trend wurde für PASI 90 beobachtet, den 73,8% zum Zeitpunkt der Verlängerung des Dosierungsintervalls erreicht hatten. Nach einer leichten Verschlechterung nach 9 Monaten (66,7%), stieg dieser Wert auf 85,7% der beobachteten Fälle nach 12 Monaten. Hinsichtlich der minimalen Resterkrankung, PASI ≤ 1, hatten 91,8% der Patienten zum Zeitpunkt der Verlängerung des Dosierungsintervalls PASI ≤ 1. Nach einer anschließenden Verschlechterung nach 6 Monaten (75%) erreichten 92,9% nach 12 Monaten PASI ≤ 1. Insgesamt hatten von den 56 Patienten, die PASI ≤ 1 zum Zeitpunkt der Verlängerung des Dosierungsintervalls erreicht hatten, drei das Ansprechen nach 3 Monaten, sieben nach 6 und 9 Monaten und acht nach 12 Monaten (14,3%) verloren (Tabelle 1, Abbildung 1a).

Zehn Patienten (16,4%) brachen die Therapie mit verlängertem Dosierungsintervall ab und kehrten zur herkömmlichen Dosierung zurück. Insgesamt wurde die Therapietreue bezüglich der Behandlungsmodulation mittels Kaplan‐Meier‐Analyse nach 1 Jahr auf 70% geschätzt (Tabelle 1, Abbildung 1b).

Hinsichtlich des durchschnittlichen PASI gab es in der Prä‐/Post‐Analyse nur einen Unterschied zwischen Behandlungsbeginn und Zeitpunkt der Verlängerung des Dosierungsintervalls (12,2 vs. 0,7, p < 0,001), jedoch keine signifikanten Unterschiede zwischen Beginn der Verlängerung des Dosierungsintervalls und den nachfolgenden Zeitpunkten (3, 6, 9, 12 Monate) (Tabelle 1).

## DISKUSSION

Nur bei einem kleinen Anteil unserer Psoriasis‐Patienten, die mit Biologika behandelt wurden, wurde eine Verlängerung des Therapieintervalls vorgenommen. Andererseits erfolgte die Therapieänderung durchschnittlich nach beinahe 2‐jähriger Behandlung. Dadurch verringerte sich die Anzahl der Kandidaten unter unseren Psoriasis‐Patienten aufgrund der verringerten Anzahl beobachteter Fälle bei extremen Zeitpunkten.

IL‐17‐Inhibitoren, insbesondere Brodalumab, waren die häufigsten Biologika, bei denen eine Verlängerung des Dosierungsintervalls vorgenommen wurde. Mögliche Gründe könnten die große Anzahl Patienten sein, die länger mit diesen Medikamenten, die früher für IL‐23 zugelassen wurden, behandelt wurden, und die hohe Anzahl geplanter Jahresdosen sein, da es neben Adalimumab das einzige Medikament mit einer therapeutischen Latenzzeit von weniger als 1 Monat ist. Ein hoher BMI und durchschnittlicher PASI am Beginn der Behandlung wirkten sich negativ auf die Eignung als Kandidat für eine Verlängerung des Dosierungsintervalls aus. Der durchschnittliche PASI zum Zeitpunkt der Verlängerung des Dosierungsintervalls war sehr niedrig, wobei mehr als 70% PASI 90, allerdings nur ein geringer Anteil vollständige Läsionsfreiheit (27,9%) erreicht hatten. Dieser Befund steht in Bezug zum retrospektiven Charakter der Studie, in die ohne definierte klinische Strategie jeder Patient eingeschlossen wurde, bei dem das Dosierungsintervall verlängert wurde.

Im Verlauf des Jahres nach der Verlängerung wurde zwischen 6 und 9 Monaten allgemein eine geringfügige Verschlechterung der Outcomes beobachtet. Allerdings kehrten, bei einer geschätzten Therapietreue von 70% nach einem Jahr, nur zehn Patienten zur Standarddosierung zurück, und es wurden keine wesentlichen Unterschiede zwischen den Behandlungen festgestellt. Das Erreichen einer minimalen Resterkrankung blieb für alle Endpunkte über 90%, wobei wir allerdings eine allgemeine Verschlechterung des Krankheitsbildes während des verlängerten Dosierungsintervalls bei mindestens 14% der Patienten berichten müssen.

In der Literatur konzentrieren sich die meisten Studien aus der Praxis auf die Modulation von Anti‐TNFα‐Therapien (Adalimumab, Etanercept, Infliximab), während Daten zu Secukinumab und Brodalumab nur aus Studien der Phasen II/III abrufbar sind.^11^, ^14^, ^15^ Die Auswahl der Kandidaten unter den Psoriasis‐Patienten für eine therapeutische Deeskalation unterschied sich bei den verschiedenen Arbeiten. Manche erforderten das Erreichen von PASI 100, andere erforderten nur PASI < 8 und wieder andere wie Ataly et al. verwendeten PASI < 5 und DLQI < 5.^16^, ^17^, ^18^

Diese Outcomes wurden für 3 Monate bis über ein Jahr aufrechterhalten, bevor das Dosierungsintervall verlängert wurde.^11^ In der Literatur fanden sich unterschiedliche Strategien zur Verlängerung des Dosierungsintervalls: Adalimumab wurde alle 3 bis 6 Wochen verabreicht, Infliximab alle 9 bis 11 Wochen, Ustekinumab bis zu eine Injektion alle 24 Wochen, Brodalumab von 4 bis 6 Wochen.^11^ Nach der Deeskalation war Adalimumab bei 100% der von Fotiadou et al. analysierten Patienten wirksam, aber nur bei 44% der von van Bezoojen et al. untersuchten Patienten.^17^, ^19^ Ebenso zeigten Etanercept eine Wirksamkeit von 23% bis 100%, Infliximab von 75% bis 100%, Ustekinumab von 22% bis 85% und Secukinumab von bis zu 85,7%. Brodalumab erhielt nach 52 Wochen einen PGA (*Physician Global Assessment*) von 0/1 bei 12,3% beziehungsweise 5,3% der Patienten aufrecht, die eine Verlängerung des Dosierungsintervalls auf eine Injektion alle 4 beziehungsweise 8 Wochen vorgenommen hatten.^11^ Auch die Beurteilung der Verschlechterung des Hautzustands unterschied sich in den verschiedenen Artikeln von Verlust von PASI < 8 bis Verlust von PASI 100.^17^, ^19^ In einer Übersichtsarbeit sprachen sich Michielsen et al. aufgrund eines Anstiegs der berichteten unerwünschten Ereignisse gegen eine Verlängerung des Dosierungsintervalls von Infliximab aus. Im selben Artikel wurde ein unwesentlicher Anstieg von Anti‐Drug‐Antikörpern bei Patienten berichtet, bei denen das Dosierungsintervall von Adalimumab verlängert wurden. Dies steht im teilweisen Gegensatz zu Aussagen, die vor kurzem von unserer Gruppe gemacht wurden.^5^, ^11^ Wir unterbreiteten den Vorschlag, geeignete Patienten für eine Verlängerung des Dosierungsintervalls unter Biologikum‐naiven Superrespondern (pASI 100 nach 6 Monaten) mit Aufrechterhalten des Ansprechens für mindestens 6 Monate und ohne Merkmale wie Beteiligung der Gelenke oder spezieller Lokalisationen, hohem BMI und metabolischem Syndrom auszuwählen.^5^

Vor kurzem schlugen Gisondi et al. eine Bedarfsbehandlung für Risankizumab bei 64 Patienten vor, die vollständiges Ansprechen Patient, der (pASI 100) nach den ersten drei Injektionen (Induktionsphase) berichtet hatten. Die Patienten nahmen die Verabreichung des Medikaments erst bei Auftreten eines PASI > 1 wieder auf. Die Durchschnittszeit zwischen drei und 14 Injektionen betrug 32 Wochen verglichen mit zwölf beim herkömmlichen Schema, und stieg bei den nächsten beiden Injektionen auf 34 beziehungsweise 39 an.^12^

In ihrer Praxiserfahrung mit IL‐17‐Inhibitoren berichteten Schots et al. eine mediane Zeit von 69,7 Wochen bis zu einer Modulation oder Modifizierung der Therapie, ohne Unterscheidung zwischen Deeskalation, Eskalation, Umstellung oder Unterbrechung der Arzneimittelgabe. In ihrer Studie wurde die Therapie bei insgesamt 18,7% der Patienten (25) angepasst: bei 16 erfolgte eine Eskalation und bei neun (6,7%) eine Deeskalation der Therapie. Von den neun deeskalierten Patienten kehrten zwei zum herkömmlichen Schema zurück.^12^ Die Autoren führten in ihrer Arbeit die Möglichkeit ein, geeignete Patienten für eine Therapieanpassung nicht aufgrund ihrer klinischen Merkmale oder ihres Ansprechens auszuwählen, sondern anhand des therapeutischen Drug Monitorings mittels Blutprobenanalyse. Bei einem Patienten mit einer Konzentration des Biologikums im Blut über dem Normbereich könnte, bei gleichzeitigem dauerhaftem therapeutischem Ansprechen, die Deeskalation eine geeignete Strategie darstellen.^13^

Klinische Kriterien für ein Rezidiv nach einer Verlängerung des Dosierungsintervalls wurden einzig für Adalimumab identifiziert, konkret hoher BMI, während männliches Geschlecht und die Eigenschaft eines Superresponders mit dem Erhalt eines verzögerten Therapieschemas assoziiert sind.^11^, ^20^, ^21^

Die in einigen Artikeln berichteten durchschnittlichen Kosteneinsparungen reichen von 13% bis 19% jährlich.^11^ Diese Ergebnisse erscheinen niedrig im Vergleich zu den Einsparungen, die durch die Verlängerung des Dosierungsintervalls von Dupilumab bei der Behandlung von Patienten mit schwerer atopischer Dermatitis erreicht werden und die laut aktuellen Schätzungen von Spekhorst et al. über 30% betragen.^7^

Die Limitationen unserer Erfahrungen sowie derjenigen der präsentierten Studien umfassen die starke Heterogenität der betrachteten Charakteristika und Outcomes, die geringe Anzahl der analysierten Patienten und die inhärenten Einschränkungen von Studien aus der Praxis.

Insbesondere wurden alle Patienten, bei denen eine Verlängerung des Dosierungsintervalls erfolgte, in die Studie aufgenommen. Dies ermöglicht zwar die Identifizierung der Patientencharakteristika bei Behandlungsbeginn, schränkt jedoch Überlegungen hinsichtlich möglicher klinischer Strategien zur Patientenauswahl ein. Aus unserer Sicht könnte selbst ein Patient der zwar kein vollständiges therapeutisches Ansprechen Patient, der (pASI 100) aber eine gewisse Stabilität des Ansprechens erreicht hat, Kandidat für eine Verlängerung des Dosierungsintervalls sein.

Da in unserer Arbeit nur zwei Patienten das Dosierungsintervall von Tildrakizumab verlängerten, konnten wir zu diesem Medikament keine Schlussfolgerungen zur Anwendbarkeit dieser Strategie ziehen. IL‐23‐Inhibitoren sind in unserer Arbeit generell nur schlecht repräsentiert. Obwohl IL‐17‐ und IL‐23‐Inhibitoren auch in selteneren Varianten der Psoriasis wirksam sind, wurde das Dosierungsintervall nur bei Patienten mit klassischer Plaque‐Psoriasis verlängert.^22^

Allerdings wird in der vorliegenden Studie erstmals eine Strategie zur Verlängerung des Dosierungsintervalls bei Patienten mit einem IL‐23‐Inhibitor angewandt. Zudem stellt sie eine der größten Erfahrungen aus der Praxis zur Deeskalation mittels Verlängerung des Dosierungsintervalls von IL‐17‐Inhibitoren dar. Randomisierte kontrollierte Studien wie BeNeBio zu allen IL‐23‐ und IL‐17‐Inhibitoren und GUIDE zu Guselkumab könnten unsere Ergebnisse bald bestätigen.^23^, ^24^

### Schlussfolgerungen

Therapiemodulation, z. B. durch Verlängerung der Dosisabstände, ist bei den meisten Psoriasis‐Patienten, wenn sie über längere Zeit fast oder ganz erscheinungsfrei sind, eine effektive Strategie. Künftige Studien sollten Deeskalationsstrategien untersuchen. Ihre Ergebnisse kännten zu Kostensenkungen beitragen und das Gesundheitssystem nachhaltiger machen, ohne die Wirksamkeit von Biologika bei der Behandlung der Psoriasis wesentlich zu beeinträchtigen.

## FINANZIERUNG

Die vorliegende Studie wurde zum Teil durch UCB pharma finanziert.

## DANKSAGUNG

Open access publishing facilitated by Universita degli Studi di Torino, as part of the Wiley ‐ CRUI‐CARE agreement.

## INTERESSENKONFLIKT

Keiner.
